# Team members’ reflections in debriefings after simulated robot-assisted surgery

**DOI:** 10.1007/s00464-025-12306-y

**Published:** 2025-10-31

**Authors:** Jannie Lysgaard Krog, Lene Spanager, Doris Østergaard, Birgitte Bruun

**Affiliations:** 1 Copenhagen Academy for Medical Education and Simulation (CAMES), Center for Human Resources and Education, Capital Region of Denmark, Copenhagen, Denmark; 2 Department of Clinical Medicine, University of Copenhagen, Copenhagen, Denmark; 3 Department of Surgery, Hospital of North Zealand, Hillerød, Denmark

**Keywords:** Robot-assisted surgery, Simulation, Coordiantion, Teamwork

## Abstract

**Introduction:**

Robot-assisted surgery (RAS) challenges teamwork due to the physical separation of the console surgeon from the rest of the team. In acute situations, such as massive bleeding requiring emergency undocking and conversion to open surgery, effective coordination becomes critical. Existing research on teamwork during acute RAS is limited, and so is research on team reflections about performance, which is essential for improving it. This study aimed to explore how RAS teams reflect on coordination in debriefings following simulated scenarios of massive bleeding.

**Methods:**

We conducted a qualitative, explorative study using thematic analysis of six debriefings held after simulation-based RAS scenarios involving massive bleeding. Debriefings were audio-recorded, transcribed, and thematically analysed.

**Results:**

Three main themes were identified: (1) *Roles and responsibilities*—highlighting the mediating role of the assistant surgeon, perceptions of leadership, and the importance of trust and familiarity; (2) *Management of tasks*—describing busyness, anticipation, tone during crisis, and hesitation to act; and (3) *Management of information*—including sub-teams in the team and the need for feedback and information.

**Conclusion:**

Team reflections emphasise role clarity, leadership, anticipating, communication, and coordination in acute RAS situations. These insights are useful for planning simulation-based training that targets these aspects during emergencies in RAS.

Robot-assisted surgery (RAS) is rapidly expanding globally. Although RAS has enhanced surgical care compared with open surgery in terms of decreased length of hospital stay, reduced blood loss, and improved recovery rates [1], it challenges teamwork. In RAS, the surgeon is positioned away from the bedside and the rest of the team. Previous research reveals that especially communication in the dispersed team is challenging and that the situation awareness of the console surgeon is potentially compromised [2].

RAS is primarily used in routine situations, and crisis situations are rare. However, if an emergency arises and requires conversion to open laparotomy, the team is hindered from direct access to the patient. The undocking process requires the console surgeon to release the instruments before the team removes them, and that the robot is removed from the patient. Additionally, the surgeon must scrub before being able to perform open surgery. These actions require prompt attention, action, and effective coordination.

Research in acute RAS situations is sparse. A recent review identified only seven studies on simulated emergency undocking in RAS [3]. Studies reported that shorter conversion time resulted in fewer errors [4] and that more simulation attempts resulted in shorter conversion time [4, 5]. Studies also reported on participants’ confidence in the procedure and found that participants were more confident after simulation-based training [6]. Another of the included studies compared two teams, one of which was trained in an emergency undocking protocol between the two simulations [7]. Both teams improved non-technical skills (situation awareness, decision-making, leadership, and communication and teamwork) between the two simulations, but the intervention group scored higher. None of the included studies reported on participants’ reflections that might be relevant to know and activate for training purposes.

This study aimed to explore how RAS teams reflect on coordination in debriefings following simulated scenarios of massive bleeding.

## Methods

**Fig. 1:**
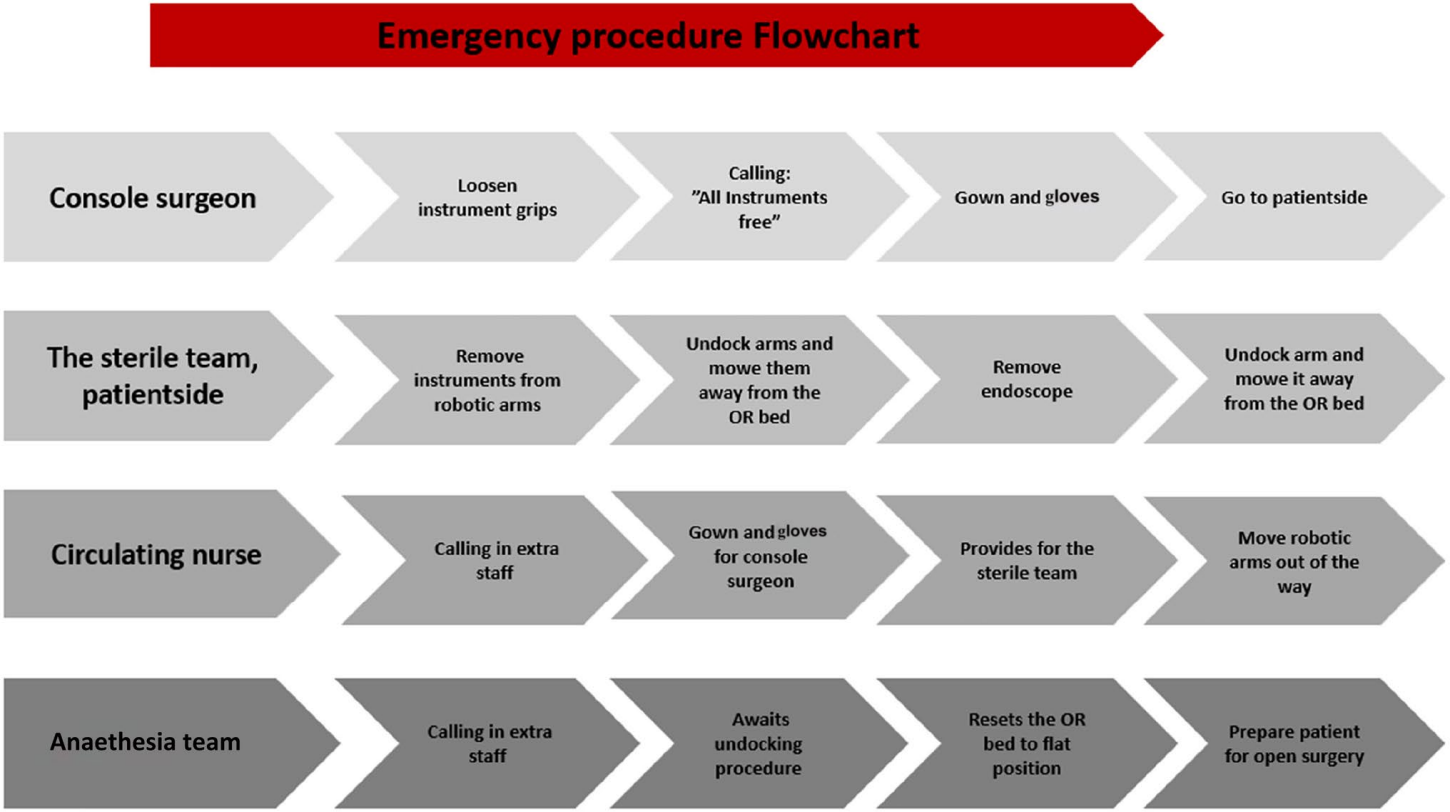
Flowchart of tasks during emergency procedures for the robot-assisted surgery team

We conducted a qualitative study of debriefings after a simulated RAS scenario that involved severe bleeding. A flowchart of tasks for each profession during the emergency undocking of the robot was available to participants in the simulation room (see Box 1 for a description of the scenario and Fig. 1 for the flowchart used by participants). Debriefings were recorded, transcribed, and analysed using thematic analysis [8]. Danish Law exempted the study from ethical review (Exemption number: H-20070513). All participants were informed about the study and that their data would be anonymised. All gave their written consent to allow the use of their data in a scientific study.

Box 1. Brief description of scenario A 72-year-old man scheduled for a heminephrectomy due to renal cancer. The medical history included hypertension and a prior appendectomy. The simulation began once adhesions had been released, and the surgical teams were instructed to prepare for heminephrectomy. During the procedure, severe bleeding occurred from a catheter positioned beneath the kidney, intentionally designed to be difficult to identify right away. In groups where an anaesthesiologist was not present, anaesthesia nurses were instructed to contact one as they would under real clinical conditions. They were then informed by phone that the anaesthesiologist was en route. The scenario concluded either when the bleeding was controlled or when the surgical team undocked the robot and prepared to perform a laparotomy.

### Setting and participants

The debriefings were held at the Copenhagen Academy for Medical Education and Simulation (CAMES) from October 2020 to February 2021.

Three teams were included from a one-day RAS team training course at CAMES, and three teams from the Robotic Surgery Department at Herlev and Gentofte Hospital, Capital Region of Denmark. The teams had participated in a simulated RAS scenario that involved massive bleeding immediately before the debriefing [9]. Teams consisted of a console surgeon, an assistant surgeon, two surgical nurses, a certified anaesthesia nurse, and, in two teams, an anaesthesiologist. In one training team, three course participants observed the scenario and participated in the subsequent debriefing. Teams from the following specialties participated: general surgery, gynaecology, urology, and anaesthesiology.

Teams from the hospital’s RAS operating section participated in a comprehensive 45 min session that included briefing on the scenario, simulation, and debriefing. Teams from the course simulated one RAS scenario earlier the same day and had 45 min for the debriefing.

Four faculty members served as facilitators in the scenarios. Two facilitators conducted the debriefings: one experienced facilitator and one facilitator who was an experienced RAS nurse or surgeon. Debriefings followed a structure of three phases: description, analysis, and application [10]. Debriefings centred on the learning objectives of the simulated scenario, which aimed to train participants in communication and teamwork, focussing on coordination and decision-making. Participants received the learning objectives of the simulations as part of the introduction to the scenario. All teams were asked to evaluate their coordination and suggest areas for improvement. Besides these foci, the contents of the debriefings were, to a high extent, formed by participant contributions. Debriefers’ questions were intended to stimulate reflection rather than provide feedback.

### Data collection and analysis

Debriefings were audio-recorded and transcribed by JLK and analysed by JLK and BB. We used thematic analysis to explore team members’ perspectives on coordination. An extensive part of the three debriefings from the team training course focussed on technical and medical issues related to the robot and the patient’s treatment. In accordance with the study’s aim, we only analysed the parts of the debriefings that related to the learning objectives.

The thematic analysis followed Braun and Clarke’s model in six steps: (1) familiarising with the data, (2) generating initial codes, (3) searching for themes, (4) reviewing potential themes, (5) defining and naming themes, and (6) reporting [8].

After completing step (1), debriefings were coded in three cycles until no new topics were identified in the data. JLK and BB initially coded all transcripts. After the first coding cycle, the codes were discussed, and the data were recoded using new codes created from the original data. The two authors again discussed the codes and developed a final coding scheme. Finally, JLK coded the six debriefings using the coding scheme.

BB and JLK searched for themes in the coded data and presented them to DO and LS. Ultimately, there was consensus amongst all authors that three themes were productive for analysing RAS teams’ perspectives on coordination through the debriefings.

### Reflexivity

The authors of this paper are all researchers with experience in medical education, representing diverse backgrounds and expertise. Three authors are medical doctors; two have experience in anaesthesiology, and one is a general surgeon. One author is an anthropologist. DO is a medical education and simulation professor and a former director of a large simulation unit. The authors’ diverse backgrounds enabled them to bring different perspectives to the data.

## Results

Thirteen doctors and 20 nurses with a mean time since graduation of 17.9 years (0–43 years) participated in six debriefings. The participants had a mean of three and a half years of RAS experience, ranging from novices to 12 years.

Debriefings of teams enrolled from surgical departments ranged from 16 to 25 min in duration. During these debriefings, one of the facilitators conducted a brief description phase to ensure as much time as possible for the analysis and application phases. Debriefings of teams enrolled from the team training course ranged from 44 to 51 min.

### Thematic analysis

Through thematic analysis, we created three themes: (1) roles and responsibilities, (2) management of tasks, and (3) management of information.

The three themes are closely related yet analytically separated. Each theme is delineated in the following sections. Representative quotes supporting each theme are summarised in Table 1.

**Table 1:** Quotations from team members supporting the three themes

Theme/subtheme	Quote
**Roles and responsibilities**	
The mediating role	… the one standing by is to some extent more engaged … with the anaesthesia – than the one in the console, who is somewhat detached from what is happening in the room […] I can look at the anaesthesia and sort of look at… how they look and what they think about [the situation] and kind of try to communicate with them. In that way, I actually believe that the role becomes some form of link [between the console surgeon and the anaesthesia] *Assistant surgeon with console experience 5*
	For me, it is incredibly important, that there is at least communication with you [assistant surgeon]. I accept that there might not be communication with ‘console surgeon’ besides hearing him say “I am working on it” or “I have it under control. I am suturing” or something. But that you can turn your head and look at me, that means that I know you have heard what I am saying *Anaesthesiologist 5*
The leader role	…If it is a crisis situation, then I believe… that some console surgeons would have said something to the anaesthesia directly… It depends on what type of person you are *Assistant surgeon 5*
	I personally find it difficult to take that [leading] role myself in real life *Console surgeon 2*
Setting the tone	If a complication arises, you won’t have to worry because she [a certain console surgeon] knows how to handle the situation. If she sits in the console and she is calm, then I don’t have any worries *Assistant surgeon 2*
	When we have a problem, we keep calm in the room. Not panicking *Console surgeon 2*
Trust and distrust	I really trust ‘console surgeon’. We have been working together for many years *Assistant nurse 2*
	Yes, so I wanted to check if everything was going well, because I did not really trust my surgeon. (Everyone laughs). I am not thinking about the surgeon in person, but it could be that something [blood] was somewhere else [than in the scope of the camera]. That is the disadvantage of robot-assisted surgery. There could easily be something down there, that you cannot see *Anaesthesiologist 3*
**Management of tasks**	
Busyness	I think it’s the circulating nurse who carries the heavy load when there’s a need to convert. You just have to be in 20 places at once *Anaesthesia nurse 1*
	…It’s the thing about staying ahead. Something was bleeding, and then I thought, it might bleed quit a lot in that suction and then maybe being prepared, if something will happen, right? *Circulating nurse 5*
Necessity of thinking and acting ahead	I just noticed that he [patient] was quite circulatory affected. His heart rate started to increase a bit. And you know, that when they have a hole on a large vessel, then it is best to have your anaesthesiologist in the room *Anaesthesia nurse 3*
	You are standing there and evaluating, what can I do, what are they doing, and what [utensils] can they receive *Circulating nurse 4*
Tension between intention and possibility to act	You actually have some time, where you cannot help with anything at all. It is a quite frustrating situation to be in, because you can see how busy it is at the bedside… *Console surgeon 4*
Hesitation to act	We are not so used to bleeding. So, I think you had to sort of prepare yourself to you saying things all the time because it’s very rare that we deal with bleeding in our area. So mentally, we were a little bit behind, realising that this is about bleeding *Assistant surgeon 3*
**Management of** **information**	
Team segmentation	…we are separated by the green drape and something surgical is going on at one side and something anaesthesiologic on the other side. And sometimes it can be difficult in all ways to get across that drape… *Anaesthesiologist 5*
	When we enter such a crisis situation, you are on your area, and I am on mine. So, it [the team] becomes actually two. You focus on the bleeding, and of course also on the patient. I focus on getting the blood pressure up and on the patient. But it is kind of two teams in that moment *Anaesthesia nurse 4*
	…it is about getting control of the situation and getting it without disturbing too much… *Anaesthesiologist 5*
Need for information and feedback	The console surgeon had to speak up in the room again. “No one is saying anything!” When he said “do we all agree, that we will soon have to convert?” It was completely silent *Observer 4*
	You get so caught up in your own task that you completely forget [to answer the console surgeon] *Assistant nurse 4*
	It is very nice that you [anaesthesia] keep giving some status on the patient. Because we are busy. We focus on stopping the bleeding, surgery etc. We don’t know how the patient is doing *Console surgeon 3*
	…I believe, we all knew what was about to happen. But when you do not stand beside each other, you miss that feedback *Assistant nurse 4*

### Management of roles and responsibilities

This theme describes how team members reflect on their roles and responsibilities. The following subthemes were created: the mediating role; the leader role; setting the tone; and trust and distrust.

Participants described the challenges and expectations associated with their role, as well as their perceptions and evaluation of their own and other team members’ responsibilities.

Five teams discussed how the surgeon’s physical separation from the bedside and anaesthesia made communication more challenging. They stressed that the assistant surgeon had an important mediating role between the console surgeon and the anaesthesia team, e.g. by repeating information. One assistant surgeon reflected on the role and suggested that they, in the future, would pass on information from either the anaesthesia team or the console surgeon instead of being the endpoint of the information. A console surgeon from another team also requested this action. More teams found the assistant surgeon’s mediating role unique for RAS. An assistant surgeon with console experience explained that the mediating role of the assistant surgeon improved communication with the anaesthesia sub-team. In addition, she felt more engaged with the anaesthesia nurse when positioned next to them rather than being detached from events in the room when sitting in the console. The assistants noted that they were positioned to see the anaesthesia nurse’s facial expressions and could interpret how he felt about the situation.

The anaesthesia personnel agreed that communication with the surgical team primarily occurred through the assistant surgeon. One anaesthesiologist agreed that the assistant surgeon’s ability to look towards the anaesthesia whilst communicating was essential to ensure he or she was heard.

Participants also found that the role of the assistant surgeon could be challenging since communication with all team members occurred whilst the assistant’s tasks continued.

Several participants implied that they saw the console surgeon as the team leader. They saw the console surgeon as the one responsible for assigning tasks. Participants relied on the console surgeon to decide whether to convert to open surgery and valued the console surgeon’s efforts to involve the team in making the decision. Some participants referred to the console surgeon’s personality, preferences, and team culture as defining the team-leading role. Team members valued clear leadership and direct communication with the anaesthesia team. However, some console surgeons expressed discomfort with taking the leadership role in the team. They did not elaborate on this during debriefings.

Several teams noted how the surgeon’s tone influenced them. More experienced teams explained that knowing the console surgeon well and the surgeon’s reactions in acute situations made them believe the console surgeon could handle the situation efficiently. They also explained how the console surgeon’s calm tone spread to the rest of the team. Console surgeons explained that staying calm and maintaining a quiet atmosphere was a key focus for them during the acute situation.

Some teams found that familiarity had a positive influence on teamwork. Knowledge of co-team members’ qualifications and familiarity with the procedure were highlighted as important for good performance during undocking. Some directly expressed that their knowledge of the console surgeon made them trust him to manage the bleeding. Less-experienced teams highlighted that they believed performance could be enhanced by knowing each other better in the team.

In contrast, both anaesthesiologists expressed distrust in the information from the console surgeon when they reported that the bleeding had stopped. An anaesthesiologist explained how blood could be hidden outside the camera scope and implied that she could not be certain that the surgeon had looked for it.

### Management of tasks

Task management focuses on tasks within the team and has the following subthemes: busyness; necessity of thinking and acting ahead; tension between intention and possibility of acting; and hesitation to act.

Team members agreed that the circulating nurse was particularly busy during undocking, performing multiple tasks, including retrieving instruments for the assistants, assisting the surgeon with the surgical gown, and removing the robotic system from the bedside. Circulating nurses described the challenge of prioritizing and executing many tasks simultaneously. The challenge arose because of the time pressure in the situation and difficulties coordinating tasks with other team members.

Surgical nurses emphasised the necessity of thinking and acting ahead during the procedure. They were aware of potential actions needed based on knowledge about information in the patient’s medical journal (e.g. previous operations), bleeding, and talk about conversion, which led to proactive actions. The anaesthesia nurse felt the same obligation to be ahead of the situation.

More participants stated that thinking ahead is more critical in RAS than in other types of surgery.

One challenge in managing tasks was the tension between wanting to support other team members and the ability of those team members to contribute to task management. One console surgeon explained how it was frustrating not being able to help because the robot was not yet removed, leaving no space for her to contribute to the work at the bedside.

Less-experienced team members reflected on their hesitation to act in the acute phase. They described how the knowledge of a flowchart regarding emergency undocking not only benefited their task execution but also delayed them as they mentally went through all members’ tasks before fulfilling their own. One experienced assistant surgeon also mentioned that she felt that she was lagging behind in the simulation when the bleeding started because she rarely handled acute situations in her daily work.

### Management of information

This theme includes discussions on how the team communicates and how team members share and convey their knowledge, observations, and insights. Subthemes include team segmentation and the need for information and feedback.

Most teams mentioned that they saw an anaesthesia and a surgical sub-team. However, this division into sub-teams was addressed in very different ways. Some teams discussed how the drape could be a barrier to information exchange. The general understanding within the teams was that the two sub-teams were occupied with solving two different tasks (stop the bleeding and stabilise the patient) and that it was difficult to be informed of what was going on on the other side of the drape. The anaesthesia personnel especially expressed that they needed to be in contact with the rest of the team.

Some team members expressed ambiguity about providing enough information for the console surgeons to fulfil their tasks whilst trying not to disturb them too much. Participants from both sub-teams were particularly cautious about disturbing the console surgeon. However, some team members also expressed an ambiguity in trying not to disturb too much, but still trying to get sufficient information to fulfil their tasks during the acute situation.

One team noted that a circulating nurse actively offered to assist the anaesthesia nurse in the acute situation. The anaesthesia nurse remarked that it was only so because the circulating nurse was experienced. He also emphasised that the gesture made him calmer and feel less alone with his tasks.

Focusing on stopping the bleeding prevented the surgeon from attending to the events unfolding in the room. One console surgeon expressed gratitude for receiving unrequested updates on the patient’s status during the acute phase of the scenario. An observer noted that the console surgeon sometimes received no feedback on requests to the team, often having to ask for confirmation that their request had been heard. An assistant nurse said he forgot to answer because he got caught up in his tasks. He explained that the feedback was missed because the team members were positioned away from each other. An assistant surgeon suggested that the person requesting or sharing information should explicitly ask for feedback.

## Discussion

We conducted an explorative study of RAS debriefings, and three themes were created addressing topics that teams talk about after a simulated scenario with massive bleeding: (1) roles and responsibilities, (2) management of tasks, and (3) management of information.

In the following sections, we address most of the subthemes from the analysis.

### Roles and responsibilities

#### The role of the leader

The team members considered the console surgeon to be the team leader. However, not all console surgeons were comfortable in this role. In RAS, the console surgeon is used to taking on leadership behaviours, such as giving instructions [11]. However, as RAS is typically a routine procedure, the console surgeon will not usually need to take on an explicit leadership role. Literature suggests that the console surgeon has the overall responsibility for the team’s tasks during acute situations [5]. Participants in our study generally agreed with this statement.

#### The role of the assistant surgeon

Our teams identified the assistant surgeon as the team’s central mediator of information. This finding is supported in an observational study conducted by our group [9]. In the literature, the assistant surgeon’s role has been found to be unique in terms of communication, as reviewed by Britton et al. [12]. They found that the assistant surgeon’s communication skills referred to their ability to effectively communicate whilst making quick decisions and multitasking. To effectively fulfill this mediating role, assistant surgeons must maintain situation awareness, but our study of debriefings illuminates how knowing other team members’ roles and tasks may be just as vital.

#### Trust and familiarity

In our study, there was a general agreement amongst our participants that working with familiar team members would positively impact teamwork. A recent study has reported that team familiarity and mutual trust seem to predict perceived team effectiveness in OR teams [13]. Another study conducted observations and interviews of orthopedic surgical teams and found that familiarity and personal knowledge of one another created an atmosphere of fellowship and safety [14]. Surgery time has been shown to decrease by 40 min when the surgeon has at least 40 surgeries with the same team members, compared with no previous collaborations [15]. In RAS, higher familiarity has been associated with less inconvenience in fulfilling the task (repeated requests, requests requiring further clarification, or resulting in frustration) [16], whereas lower team familiarity has been associated with interruptions during surgery [17]. However, surgical teams are often unstable, making team familiarity hard to achieve.

### Management of tasks

#### Anticipation

In our study, thinking and acting ahead were emphasised as more crucial in RAS than in other types of surgeries, especially by surgical nurses. Mitchell et al. [18] argue that when the assistant nurse is one step ahead of the surgeon, the circulating nurse must be two steps ahead to avoid the surgeon waiting and to maintain the procedure’s flow. The increased need for anticipatory thinking in RAS has been attributed, in part, to the technically challenging tasks [19]. For instance, when changing instruments during other types of surgery, the leading surgeon may be able to manage the change independently, possibly with assistance from an assistant. In RAS, assistants must remove the instrument from the robot arm before inserting another instrument to allow the console surgeon to proceed with the procedure. The anticipatory behaviour by the surgical nurses may reduce delays in the console surgeon’s tasks, thereby increasing efficiency [19].

The participants touched upon the console surgeon’s loss of situation awareness during RAS. Other studies have also raised the concern of possible loss of the console surgeon’s situation awareness [20, 21]. Strategies to maintain situation awareness include communication from the team [22]. Ahmed et al. conducted an observational study describing how the team’s anticipation (tasks performed without or before verbal requests from the surgeon) was positively associated with the console surgeon’s situation awareness [23]. Our study findings support the claim that the console surgeon needs information from the team, and we suggest that the assistant surgeon, via the mediating role, might be the most important contributor to maintaining the console surgeon’s situation awareness through communication.

#### Tone of voice during crisis

More experienced team members commented on the effects of the console surgeon’s tone. Console surgeons were aware of the importance of maintaining a calm atmosphere and that other team members adapted to it. Less-experienced console surgeons may not have the mental capacity to think about how they influence the atmosphere during an intense simulation. In other types of surgery, team members also agree that they listen to the tone of the leading surgeon [24] and tone-setting is considered an essential part of the leading surgeons’ role [25] even though they may not be aware of it themselves [26]. However, other team members can influence and change the tone in the OR [25]. A scoping review on emotions and communication in the OR suggests that teams expect team leaders to control their emotions and remain positive [27]. Otherwise, the team leaders’ communication style might inhibit speaking up from other team members, potentially compromising patient safety [27].

#### Hesitation to act

Interestingly, both less-experienced and more-experienced team members mentioned that they hesitated to act during conversion. Although hesitation to act for the console surgeon was not mentioned in debriefings, we observed during simulations that some console surgeons hesitated in acute situations, which may relate to difficulties in changing from a flat hierarchy to a more hierarchical structure, but also challenges in determining when to make this transition and step up as a team leader.

We interpret the hesitation to act as an expression of insecurity in the situation, as RAS teams rarely experience emergency conversions. Protocols are proposed to help clinicians in these situations by outlining the role of team members [28, 29]. Some protocols do not include the anaesthesia personnel [6, 29]. In our opinion, the entire team should be included in such protocols to emphasise the importance of teamwork across specialities to ensure a good outcome for the patient. Simulation training of emergency conversions improves team performance in terms of conversion time [4] and enhances the confidence of team members [6, 30]. Although protocols help teams to improve undocking in emergencies, they can only do so if team members are familiar with them, as there is no time to look them up in an acute situation.

### Management of information

#### Team segmentation

Especially, anaesthesia team members indicated that the onset of bleeding seemed to initiate the division of the team into two sub-teams. Our findings are supported by a study on anaesthesia personnel’s perception of teamwork during RAS, where participants described feeling excluded from the team [31]. Although the team members were working towards the same goal, the tasks were distinctly different for the surgical and anaesthesia sub-teams. It appeared that this also had an impact on how teams shared information. More team members considered avoiding information overload to accommodate the console surgeon’s need to concentrate. We can think of three reasons why team members could be uncertain about how much information to share during RAS. First, it may be caused by a lack of visual contact and nonverbal feedback mechanisms, which make the sender unsure whether the information is welcomed. Additionally, it may stem from a lack of understanding about other team members’ roles and information needs. Finally, sharing information might be a strategy for requesting information as well.

#### Need for information and feedback

In an acute situation, the surgical team focuses on the surgical tasks—in our scenario, to stop the bleeding. Anaesthesia personnel rely on information about the surgical plan whilst resuscitating the patient. The moment the surgeons perform laparotomy, intra-abdominal pressure drops, which may cause the blood pressure to decrease. Therefore, the anaesthesia depends on information from the console surgeon regarding the status of the bleeding to provide adequate resuscitation of the patient. Participants in simulated crisis situations during non-robotic surgery have also highlighted the importance of sub-teams communicating clearly with one another to improve teamwork [32]. Gjeraa et al. found that thoracic surgery teams described how close collaboration between sub-teams was required during problem-solving [24]. Gaba et al. stress the importance of training each sub-team in technical, cognitive, and social skills that are relevant to their domain in crisis management [33]. They propose that sub-team training allows for discussion of other disciplines, providing an understanding of other sub-teams’ views on a particular situation. On the other hand, they also find that interprofessional team training is essential, as it enhances understanding across disciplines. Preferably, both types of training should take place [33].

### Strengths and limitations

A strength of this study is that it incorporates perspectives from the entire RAS team. The debriefing setting ensured that team members could elaborate on perspectives regarding their own and others’ roles.

No teams specifically mentioned the term coordination during the debriefings, despite debriefers explicitly using the term and asking questions about it. With hindsight, we can see how teams instead described their observations and concerns in a vocabulary that has similarities with the terminology of non-technical skills in behavioural marker systems [34–36].

The simulation prior to the debriefing may have directed the participants’ attention towards the terminology of the categories of non-technical skills, rather than focussing on the term coordination. CAMES is a regional simulation centre responsible for compulsory simulation-based team training courses for all internship doctors, surgeons, anaesthesia nurses, and anaesthesiologists in the Capital Region of Denmark. The association might have led some participants to predict a crisis situation. In a real-life setting, team members would call for assistance from colleagues during an acute situation. This was not an option in our simulation, leaving some team members with many tasks to fulfil. Although we believe it is realistic for the circulating nurse to be busy during an emergency undocking, it may be exaggerated in the simulation, leading to excessive attention in the debriefing.

For logistical reasons, teams could not repeat the scenario, which hindered the possibility for the teams to iterate on experiences from the scenario and the following discussions in the debriefings.

This study included a small sample size and was limited to participants from Denmark, where the hierarchy in acute care teams is flat. This affects how team members work together, e.g. the flat hierarchy might allow the assistant to take on more responsibility as well as how teams perceive and discuss teamwork and leadership.

### Implications for practice and education

This study extends the research on teamwork in acute care teams by adding RAS team members’ reflections in a debriefing following a simulation involving massive bleeding. The results provide insight into the issues teams encounter in acute situations, and therefore, insights into the aspects that teams should train to enhance their performance during such procedures.

Simulated crisis scenarios in both non-robotic and RAS settings have been shown to improve non-technical skills [7, 37]. However, as for previous studies in RAS, studies within interprofessional simulated non-robotic surgery do not report on the role specific to each team member [38]. Our study emphasises that team members have different roles in the RAS team. This knowledge should be taken into consideration when planning team training for RAS teams as well as when training team members individually. This study also emphasises that the role of each team member during emergency situations can be managed in many different ways, with implications for coordination and patient safety. Future research should seek to understand team members’ own strategies for improving coordination within the team, which could inform training sessions.

In conclusion, three themes were created: (1) roles and responsibilities, (2) management of tasks, and (3) management of information. Team reflections highlight the importance of training that targets role clarity, leadership, anticipation, and communication during emergencies in RAS. These insights are useful for planning simulation-based training that targets these aspects during emergencies in RAS. The assistant surgeon plays a pivotal role in mediating information due to the physical separation of the team.
